# Hemolytic anemia associated with intravenous immunoglobulin in Kawasaki disease

**DOI:** 10.1186/s12887-024-04546-z

**Published:** 2024-01-20

**Authors:** Eun Jung Cheon, Jun Suk Oh

**Affiliations:** 1 Department of Pediatrics, Chungbuk National University Hospital, Cheongju-si, Chungcheongbuk-do Republic of Korea; 2 Department of Pediatrics, Konyang University Hospital, Daejeon, Korea Republic of Korea

**Keywords:** Kawasaki disease, Intravenous immunoglobulin, Hemolytic anemia

## Abstract

**Background:**

The administration of high-dose intravenous immunoglobulin (IVIG) is a standard treatment for the management of Kawasaki disease (KD). IVIG is known to be a highly effective and safe treatment.

**Case presentation:**

We report the development of hemolytic anemia in seven children receiving repeated doses of IVIG. The children were aged 3–44 months and included 4 girls and 3 boys. All children received 10% IVIG and a second course of immunoglobulin because they did not respond to the first course of immunoglobulin. Two received high-dose aspirin (50 mg/kg), and five received low-dose aspirin (5 mg/kg). Two patients required additional methylprednisolone pulse therapy (30 mg/kg) after the second dose of immunoglobulin, and three patients received oral prednisolone therapy for defervescence. Three patients showed coronary artery dilation during hospitalization and normalized within two months. Pretreatment hemoglobin averaged 11.3–14.2 g/dL, and post-hemolytic anemia hemoglobin ranged from 7.4 to 9.6 g/dL, with a difference of 1.7–6.8 g/dL. Reticulocytes were increased to 3.3–13.2%. Peripheral blood smears showed normochromic normocytic anemia, and anisopoikilocytosis. All children were positive for warm-type antibodies with IgG+, C3d- in direct antiglobulin test, and the blood group was A + in five and B + in two. None of the patients received immunomodulatory therapy or red blood cell transfusions. They were followed for a year and all recovered.

**Conclusion:**

Especially, in non-O blood group KD patients who are refractory to initial IVIG and require a second dose of IVIG or 10% formulation the possibility of immune hemolytic anemia should be carefully considered, and close follow-up should be maintained after therapy.

## Background

Since the development of highly-purified intravenous immunoglobulin (IVIG) preparations in the 1980s, a new unmodified preparation known as native IVIG has been developed. This is now the standard of care in replacement therapy for patients with primary immunodeficiency and has been widely used as a treatment modality for various clinical conditions, providing immunomodulatory and anti-inflammatory effects [1]. IVIG therapy is the standard treatment for Kawasaki disease (KD) to reduce the risk of coronary complications [2]. IVIG’s efficacy be related to its neutralizing antibody activity against inflammatory cytokines and bacterial enterotoxins; [3] infused antibodies are also thought to prevent immune-mediated damage blocking cytotoxic T-lymphocyte recognition of infected cells. IVIG has been shown to reduce endothelial cell death by neutralizing the effects of cytokines, blocking the response of endothelial cells to cytokines, or blocking the production of cytokines and growth factors [4, 5].

IVIG is generally safe and well tolerated, but it may be associated with certain side effects. Common side effects of IVIG may include mild to moderate reactions at the infusion site, such as pain, swelling, or redness. Potentially serious side effects of IVIG include allergic or hypersensitivity reactions [6]. These reactions can range from mild allergic symptoms such as rash or itching to more severe reactions such as difficulty breathing, tightness in the chest, or anaphylaxis, a severe and life-threatening allergic reaction [7]. It is important to note that severe allergic reactions are rare but can occur, especially in individuals with a history of allergic reactions to IVIG or other blood products. In addition, IVIG may rarely be associated with certain systemic adverse effects, such as blood clotting disorders, kidney problems, or aseptic meningitis [8]. These adverse reactions are relatively rare, but should be monitored.

IVIG-induced hemolysis is a rare but known complication. However, the mechanism is not fully understood. Previous studies have shown that approximately 2.5% of KD patients receiving IVIG may develop IVIG-associated hemolytic anemia [9].

## Case presentation

The authors report seven cases of immune hemolytic anemia in Kawasaki disease and review the literature. In our center, we have performed a follow-up blood test 36 to 48 h after the completion of IVIG treatment in patients with KD. Earlier, we analyzed the laboratories of KD children in our center and found that the change in hemoglobin level was within 1.5 g/dL in the blood test performed 48 h after IVIG infusion. Therefore, we have been checking for hemolysis if the hemoglobin had decreased by 1.5 g/dL compared to pretreatment, they were evaluated for hemolysis. During six years, 367 patients were treated with the same product, IVIG. At our institution, a total of 588 patients were diagnosed with Kawasaki disease during the period, of which 367 patients received the 10% formulation and seven patients had hemolytic anemia. Prior to this period, the 10% formulation was not in use. During this period, the decision to use the 10% or 5% formulation for an individual patient is at the discretion of the physician. Of the 376 patients who received the 10% formulation, seven patients developed hemolytic anemia, of which 5 were type A, 2 were type B, and none were type O. None of the 226 patients who received the 5% formulation developed hemolytic anemia (*p* < 0.05). The incidence of anemia was 1.1% for all patients with Kawasaki disease and 1.9% for patients using the 10% formulation.

The children were all diagnosed with complete KD. The clinical characteristics of the children are shown in Table 1. The third patient appears to be an example of a patient who was treated for fever on day 3 and needed to be re-treated. Except patients 1 and 6, all of them had elevated AST and ALT levels with initial liver dysfunction, which returned to normal after treatment. Patients 2, 3, 4, and 6 had higher indirect bilirubin levels before the onset of hemolytic anemia. This is likely due to the fact that the more severe the degree of KD, the more severe the liver dysfunction and the higher the indirect bilirubin. Patient 6 had a decreased glomerular filtration rate (GFR) of 44 mL/min/m2 due to acute kidney injury (AKI) before treatment, but AKI improved and GFR normalized after treatment. No patient was found to have an autoimmune disorder or family history, and no patient was found to have autoantibodies at examination. Patient 2 complained of abdominal pain and had evidence of melena, so an antacid was administered and aspirin was discontinued, and the melena resolved in about two days.

**Table 1:** Clinical characteristics and laboratory findings of patients with hemolytic anemia in KD

	Case 1	Case 2	Case 3	Case 4	Case 5	Case 6	Case 7
Sex	Male	female	male	female	female	male	female
Age at diagnosis (months)	3	40	44	6	40	28	46
Height (centimeters)	65	80	100	71	98	93	96
Body Weight (kg)	7.8	18.0	17.2	9.3	16.5	13.6	14
Body Mass Index (kg/m^2^)	18.4	28.1	17.0	17.9	16.7	15.9	15.2
Initial fever duration (days)	3	4	5	5	4	7	7
Total fever duration (days)	8	7	9	8	7	10	9
WBC (/mm^3^)	17,300	11,300	8,800	9,700	14,200	8,020	10,300
ANC (/mm3)	12,802	8,972	5,830	7,207	13,374	6,600	7,750
Platelet (x10^3^)	285	316	188	180	299	523	478
CRP (mg/dL)	6.3	3.6	8.4	8.6	8.2	5.03	7.8
AST (IU/L)	30	681	48	195	189	16	54
ALT (IU/L)	31	671	73	91	192	8	57
NT pro-BNP (pg/mL)	720	1,803	1,217	3,054	342	11,576	1,874
Initial Hb (mg/dL)	11.3	12.1	11.1	10.3	14.2	11.4	12.0
Lowest Hb (mg/dL)	9.6	9.0	8.1	8.3	7.4	8.5	9.0
Reticulocyte count (%)	10.8	3.3	13.2	10.0	13.0	1.8	3.0
LDH initial (IU/L)	754	551	694	381	526	548	645
Indirect bilirubin (mg/dL)	0.36	4.49	3.63	1.81	0.53	1.11	0.8
Indirect bilirubin (mg/dL) *	0.83	1.19	2.88	0.51	0.61	1.25	1.67
Haptoglobin (65-195 mg/dL)	118	96	105	238	89	345	265
Blood transfusion	none	none	none	none	none	none	none
Additional Treatment	MP		MP	PD	PD	PD	
Coronary outcome	none	none	dilated	dilated	dilated	dilated	none
Blood group	A+	B+	A+	A+	A+	B+	A+
Antibody identified	IgG + C3d	IgG + C3d	IgG + C3d	IgG + C3d-	IgG + C3d-	IgG + C3d-	IgG + C3d-

*KD* Kawasaki disease, *WBC* white blood cell count, *ANC* absolute neutrophil count, *CRP* C-reactive protein, *AST* aspartate aminotransferase, *ALT* alanine aminotransferase, *NT pro-BNP* nitrogen terminal, pro b-type natriuretic peptide, *Hb* hemoglobin, *LDH* lactate dehydrogenase, Indirect bilirubin (mg/dL) *, Indirect bilirubin blood level in follow-up, *MP* methylprednisolone 30 mg/kg/day for consecutive three days, *PD* prednisolone 2 mg/kg/day

All patients received initial treatment for KD with 10% IVIG. They received a second dose of IVIG because their fever persisted for 36 h after completion of the IVIG infusion. Patients 2 and 5 became afebrile after the second dose of IVIG, and the remaining patients required further treatment. Patients 3 and 6 received pulse therapy with methylprednisolone at 30 mg/kg/day for 3 days, patient 1 received methylprednisolone at 15 mg/kg/day for 3 days, and patients 4 and 5 received oral steroids at 2 mg/kg/day for 14 days as an adjunct treatment for KD. The choice of adjunctive therapy was based on the judgment of the clinician. Serum haptoglobin levels in our patients were variable, and haptoglobin is not always decreased in IVIG-related hemolysis; it is an acute phase reactant and may be increased in the presence of inflammatory conditions. When adjusted for hemoglobin levels, patients 2, 6, and 7 appear to have normal/near normal reticulocyte counts. The reason for this is not clear, but it is possible that the compensatory activity of the bone marrow does not reach an adequate level in KD due to immunological reasons.

Five children were blood type A + and two children were blood type B+. All patients were positive for direct anti-globulin test (DAT) and antibodies were IgG + C3d with warm antibodies. Within 2 months, hemoglobin levels normalized and autoantibodies disappeared. Hemoglobin decreases ranged from 1.7 g/dL to 6.8 g/dL. Patient 5, whose hemoglobin reduction was 6.8 g/dL, had a decrease in hemoglobin level from 14.2 g/dL to 7.4 g/dL in 5 days. The patient had no signs of heart failure, such as an increased heart rate or enlarged heart, and the patient’s condition was tolerable, so a blood transfusion was not performed.

Six of the seven patients had normal body mass index (BMI) profiles and one was obese. The obese patient did not have more severe hemolysis than the other patients, and the hemolysis occurred after two doses of IVIG had been administered.

## Discussion

The risk of hemolysis in patients with KD who are treated with IVIG is multifactorial. The most common of these is the presence of transient, passively acquired antibodies, which are identified as positive DATs that trigger a direct antibody-mediated attack [10]. Researchers reported that anti-A and anti-B antibodies present in IVIG can cause hemolysis, highlighting the role of anti-RBC antibodies in IVIG-associated hemolytic anemia. Patients with blood groups other than O are at increased risk of developing hemolytic anemias due to their inability to neutralize plasma anti-A and/or anti-B alloantigen after IVIG infusion. Specifically, patients with blood groups A, B, or AB are at a relatively higher risk of developing hemolytic anemias, and clinicians should consider the patient’s ABO blood group before giving IVIG. Of the seven patients who used the 10% formulation and developed hemolytic anemia, five were blood type A, two were blood type B, and none were blood type O [11, 12].

Several risk factors that may contribute to the development of IVIG-related hemolytic anemia include blood type A, higher IVIG doses, and a history of blood transfusions. Daw et al. [13] reported that severe hemolysis occurred in 1.6% of patients treated with high-dose IVIG; characteristics of these patients included high-dose IVIG, female gender, blood type other than O, and underlying inflammatory disease. IVIG is a therapeutic preparation that contains a variety of immunoglobulins, including IgG antibodies, because it is manufactured by pooling plasma from thousands of donors. The anti-erythrocyte antibodies present in IVIG may inadvertently target the red blood cells of the patient when it is administered to the patient, resulting in hemolysis. Hemolytic anemia should be considered in patients with KD receiving IVIG, especially in those who require a second infusion. In our cases, all of the cases of hemolytic anemia occurred after the second infusion of IVIG for persistent fever (total dose of 4 g/kg). Previous studies have shown that hemolytic anemia is known to be dose-dependent.

The mechanisms underlying the hemolysis associated with IVIG are still not well understood. It seems clear that the passive transfer of anti-A and anti-B hemagglutinins plays an important role, but this does not explain the fact that hemolysis occurs in only a subset of patients receiving IVIG. Thus, a two-hit theory has been proposed [13]: the first hit is the passive transfer of anti-A and anti-B, and the second hit is the underlying inflammatory state that may be present in patients who receive IVIG. Anemia due to inflammation in KD and immune dysregulation in the disease are other risk factors for hemolytic anemia.

The risk of IVIG-associated hemolytic anemia may be significantly influenced by pre-existing conditions, particularly autoimmune diseases or previous sensitization to antigens. Individuals with a history of autoimmune disease may be more susceptible to immune reactions induced by IVIG [14]. These reactions may lead to hemolysis. Patient-specific factors, including genetic predisposition and immune system function, can affect response to IVIG. Individuals may be more susceptible to adverse reactions, including hemolysis, with IVIG administration due to certain genetic profiles. Research has shown that patients with existing autoimmune diseases, such as systemic lupus erythematosus (SLE) or rheumatoid arthritis, are more likely to experience adverse reactions to IVIG, including hemolytic events. For example, a study by Pendergrast et al. highlighted the potential risk of hemolytic events in patients with autoimmune diseases receiving IVIG therapy [14]. Before initiating IVIG therapy, it is important to review a patient’s medical history and assess for the presence of a pre-existing autoimmune disease. Patients with a history of autoimmune disease or known immune system disorders should be closely monitored during IVIG infusion for signs of adverse effects, including hemolysis. Clinicians should carefully evaluate the medical history of each patient and consider alternative therapies for those who may be at higher risk for side effects such as hemolysis.

Molecular mimicry is another hypothesis for IVIG-associated hemolysis. IVIG antibodies have the ability to mimic red cell surface antigens, a process called molecular mimicry [15]. When these antibodies are infused into a patient during IVIG treatment, their similarity to the antigens on red blood cells can cause the immune system to attack both the antibodies and the patient’s own red blood cells. This can result in hemolysis, the destruction of red blood cells, and subsequent hemolytic anemia. The molecular mimicry between antibodies and autoantigens, and the way in which this mimicry can trigger an autoimmune response, has been demonstrated in a number of studies. A study by Ross et al. [16] examined molecular mimicry between viral antigens and autoantigens and how this phenomenon can contribute to autoimmune disease. This study highlights the potential mechanisms that may be involved in molecular mimicry and autoimmunity, although not directly related to IVIG. The concept of molecular mimicry emphasizes the complexity of immune responses, especially when exogenous antibodies in IVIG mimic endogenous antigens. This emphasizes the need for caution and a thorough evaluation prior to the administration of IVIG to individuals who are at risk for complications due to molecular mimicry.

All of the patients who developed hemolytic anemia were treated with 10% formulation of the brand of the product. Patients using this product had a 1.9% incidence of hemolytic anemia. None of the patients who used the 5% formulation developed hemolytic anemia. There have also been reports that the risk of hemolysis varies with different IVIG products. Romberg et al. [17] found that the cone-like fractionation process was effective in reducing isoagglutinin levels compared to chromatography-based production methods. In one study, relatively higher rates of hemolysis were observed in IVIG products containing fluids stabilized with amino acids (glycine, L-proline). One strategy that has been studied to prevent hemolytic episodes is to screen donors with automated IAT and exclude donors with high anti-A titers from plasma pooling and fractionation. In one study, plasma from donors with high anti-A titers was found to have reduced agglutinin levels in this manner. The disadvantage of this approach is that 5–7% of donors are ineligible to donate blood/plasma. All of our patients have used the same brand of IVIG. The product states that the IgG is 96%, PH 4.25, and uses a Cohn-like fractionation procedure with 10% maltose as a stabilizing solution. All of our patients developed hemolysis with the 10% formulation of the same brand and none with the 5% formulation of the same brand. There have been no previous studies on the concentration of IVIG. It is difficult to know exactly why there was more hemolysis with the 10% product. The presence of high molecular weight IgG complexes in IVIG can bind to complement receptors on red blood cells, resulting in erythrocytosis and making them more susceptible to hemolysis [18]. The presence of polymers of IgG may be a more likely contributor to IVIG-associated hemolysis. The European Pharmacopoeia recommends that the polymer content of IVIG preparations should be less than 3% [19]. Dimers and multimers can act as immune complexes and bind to complement on erythrocytes. Dimeric IVIG is more likely to be enriched in idiotype/anti-idiotype complexes. It does not provide information about the blood group of the donor and the titer of the isoagglutinin. The problem is that IVIG is limited in the country today. It is difficult for prescribing physicians to obtain detailed information about each IVIG because of the relatively limited information on anti-A or anti-B titers, information on the blood donor pool, and the exact ingredients or manufacturing process. It is likely that the 10% IVIG formulation has a higher percentage of IgG dimers or polymers than the 5% formulation of the same product, but no information is available. Each IVIG product uses a different stabilizing solution. For example, Sandoglobulin is a lyophilized sucrose-stabilized IVIG preparation (CSL Behring AG, Bern, Switzerland). Dantal et al. reviewed the types of excipients used in IVIG and observed relatively high rates of hemolysis with amino acid (glycine, L-proline) stabilized liquid IVIG products [20]. A 10% maltose stabilizer was used in the product used by the patients in our report.

Van Anh et al. [21] reported that no cases of hemolytic anemia occurred with a single dose of IVIG in 496 non-obese patients, whereas two cases of hemolytic anemia occurred with a single dose in 36 obese patients. This means that at a dose of 2.7 g of IVIG per kg of lean body weight, obese patients developed hemolytic anemia. Therefore, clinicians treating patients with KD should monitor for hemolytic anemia after the second dose of IVIG, and the dose of IVIG should be adjusted based on lean body mass in patients with obesity. In our patients, only one child was obese and six patients had a normal BMI profile, and obese patients did not show more severe hemolysis. Further studies in larger populations of patients are needed to determine whether the hemolysis in obese patients is different from that in lean patients. It is important to remember that patient factors such as individual susceptibility, underlying medical conditions, and medical history may also influence the occurrence of certain side effects. Therefore, it is important to consider side effect concerns and refer to the specific product information for detailed and accurate side effect information for your particular IVIG product.

In our experience, most patients had mild hemolysis and did not require blood transfusion. All but two were on oral steroids. Hemoglobin normalized within 1–2 months and autoantibodies persisted longer. In our experience, the hemolytic anemia that occurs after IVIG treatment is usually mild and resolves without special treatment. However, in patients with the most severe hemolysis, hemoglobin levels decreased by up to 6.8 g/dL, requiring several more days in the hospital and more than one outpatient blood draw. For these children, this is a painful fact that cannot be ignored. In recent years, the next treatment options for patients who become resistant to initial IVIG therapy have included additional IVIG, infliximab, and methylprednisolone pulse therapy, but all have proven to be equivalent in preventing coronary aneurysm complications [22]. Therefore, clinicians are encouraged to choose treatment options that lower morbidity by reducing hospitalizations and side effects. Therefore, the authors suggest that if a patient has a blood type other than O and the only available IVIG is a 10% formulation, they may consider choosing a second-line therapy due to the potential for hemolytic anemia side effects.

The management of IVIG-induced hemolysis depends on the severity of the condition and the patient’s clinical presentation. When IVIG-induced hemolysis is suspected or confirmed, the following management strategies may be considered. The first step to prevent further hemolysis and to assess the patient’s condition is usually to stop the IVIG infusion. It is important to provide supportive care to manage the symptoms and complications of hemolysis. This may include monitoring vital signs, assessing oxygenation, treating fluid and electrolyte imbalances, and supporting transfusions as needed. Frequent monitoring of the patient’s clinical status, including vital signs, urine output, hemoglobin levels, and markers of hemolysis (such as bilirubin and lactate dehydrogenase), is critical in assessing the severity of hemolysis and making further management decisions. If severe hemolysis or complications have occurred, it is recommended that a hematologist or transfusion medicine specialist with expertise in the management of hemolytic disorders be consulted to guide an appropriate treatment plan. In cases of severe hemolysis, transfusion of packed red blood cells (PRBCs) may be required to treat anemia, improve oxygen-carrying capacity, and treat the clinical symptoms of hemolysis. The decision to transfuse blood products should be individualized to the patient’s condition based on hemoglobin levels, evidence of tissue hypoxia, and other clinical parameters. In rare cases of severe or prolonged IVIG-induced hemolysis, additional immunomodulatory therapies may be considered. These may include corticosteroids, intravenous immunosuppressants, or other targeted therapies such as bortezomib [21] or rituximab [22] to modulate the immune response and reduce hemolysis.

## Data Availability

Study data and materials not included in this article are available from the corresponding authors upon reasonable request.
